# Balance control during stance - A comparison between horseback riding athletes and non-athletes

**DOI:** 10.1371/journal.pone.0211834

**Published:** 2019-02-05

**Authors:** Agnès Olivier, Jean-Philippe Viseu, Nicolas Vignais, Nicolas Vuillerme

**Affiliations:** 1 CIAMS, Université Paris-Sud, Université Paris-Saclay, Orsay, France; 2 CIAMS, Université d'Orléans, Orléans, France; 3 Groupe Voltaire—Forestier Sellier, Bidart, France; 4 AGEIS, Université Grenoble Alpes, Grenoble, France; 5 Institut Universitaire de France, Paris, France; University of Minnesota, UNITED STATES

## Abstract

Horseback riding requires the ability to adapt to changes in balance conditions, to maintain equilibrium on the horse and to prevent falls. Postural adaptation involves specific sensorimotor processes integrating visual information and somesthesic information. The objective of this study was to examine this multisensorial integration on postural control, especially the use of visual and plantar information in static (stable) and dynamic (unstable) postures, among a group of expert horse rider women (n = 10) and a group of non-athlete women (n = 12). Postural control was evaluated through the center of pressure measured with a force platform on stable and unstable supports, with the eyes open and the eyes closed, and with the presence of foam on the support or not. Results showed that expert horse rider women had a better postural stability with unstable support in the mediolateral axis compared to non-athletes. Moreover, on the anteroposterior axis, expert horse riders were less visual dependent and more stable in the presence of foam. Results suggested that horseback riding could help developing particular proprioceptive abilities on standing posture as well as better postural muscle tone during particular bipodal dynamic perturbations. These outcomes provide new insights into horseback riding assets and methodological clues to assess the impact of sport practice.

## Introduction

Sport practice constraints players to manage simultaneous sources of information in order to maintain postural stability in an efficient manner. This process may be called “adaptive postural control” [1,2]. The contribution of sensory information to postural control has been showed to differ according to the sport activity [3–5] and the level of practice [6,7]. In a recent review, Paillard [8] concluded that repeated particular postures and movements, induced by sport practice, could generate robust postural adaptations. This would be especially the case when the sport practice induces a high level of postural balance during aerial and ground-contact phases, as in gymnastics. Vuillerme and colleagues [9] compared postural control of a group of expert gymnasts vs. a group of experts in other non-gymnastic sports in three standing postures of increasing difficulty: bipedal, unipedal, and unipedal with unstable support (i.e. 7 cm thick foam surface). Results showed that gymnasts had significantly less postural sway when vision was removed in unipodal tasks. Surf practice is also requiring a high level of postural abilities while standing on the surfboard. In an expert vs non-expert study, Paillard and colleagues [10] analyzed postural control in different visual conditions (open and closed eyes) and stability (static and dynamic) conditions. Postural parameters were therefore assessed by measuring the center of foot pressure displacement. The authors showed that expert surfers had better postural control and they used less visual information when maintaining posture in unstable support.

Like horseback riding, canoeing requires postural stability in a sitting posture. Stambolieva and colleagues [5] studied the postural stability of 23 canoeing and kayaking athletes vs. 15 healthy untrained subjects. The influence of two conditions of vision (open and closed eyes) and two conditions of stability (stable and foam support) on center of pressure excursions was analyzed while standing. Results demonstrated that kayaking and canoeing athletes had a better postural stability on an unstable support while standing with eyes open. Moreover, it appeared that the result of Romberg Quotients (RQ) which evaluated the contribution of vision on standing posture, showed that canoeists were more “visual-dependent” than kayakists. This may be related to the fact that canoeists are dealing with a kneeling posture during their activity. Visual dependency reflects the weight each individual assigns to visual or non-visual information during postural control [11]. In cycling, the athlete is also sitting and needs postural stability to avoid falls. Lion and colleagues [12] compared postural abilities of mountain bikers and road cyclists. They showed significant differences between groups with road cyclists being more sensitive to vision to control balance during stance than mountain bikers.

Maintaining postural stability in horseback riding is a critical constraint to ensure safety and avoid falls. Postural stability depends on the sitting posture adopted by the rider, with a leg on each side of horse, commonly referred as a ‘straddle posture’. When horseback riding, the most obvious source of information comes from the visual field. In a recent study, Olivier and colleagues [13] evaluated the relative contribution of visual information to horseback riders' postural stability (estimated from the variability of segment tridimensional position). Postural parameters were measured on an equestrian simulator for a group of expert riders and a group of club riders in four visual conditions: real-simulated ride scene, stroboscopic illumination to prevent access to dynamic visual cues, no projected scene under normal lighting, and no visual information. Results suggested that professional riders had a greater overall postural stability than club riders, mainly revealed in the anteroposterior axis. Thus, intensive training in horseback riding induces changes in postural control measured on an equestrian simulator. It might be therefore interesting to investigate the influence of this intensive training on postural control in a standing posture. Horse movements may also be considered as a source of information for the rider, inferring postural imbalance acting from the pelvis to the rider’s head [14–16]. Adapting to this instability may thus lead to specific postural skills related to vestibular, proprioceptive and cutaneous information.

Postural effects of sport have been evaluated in amateur and competitive practitioners through comparisons with control participants. These experimental designs have been performed in many sports such as dancing [17], soccer [18,19], volleyball [20], rugby [21], kitesurfing [22], or running [23]. Thus, differences between balance control during stance in athletes and non-athletes seem to have potential in elucidating the effect of horseback riding training on postural control regardless of their initial postural abilities.

Horseback riding can thus be considered as a sport activity with particular postural constraints. The subsequent scientific question is whether or not horseback riding intensively would influence postural abilities. The main hypothesis is that horseback riding athletes would exhibit a different sensory organization of balance control compared to nonathletes. To answer this question, postural stability parameters involved in center of pressure displacement have been compared for two groups, horseback riders vs non-athletes, in different experimental conditions implying vision, support stability and proprioception.

## Materials and methods

### Participants

Ten elite professional riders specialized in dressage (‘DR’ group), and twelve non-athlete women (‘NA’ group) who did not practice horseback riding, voluntarily participated in the experiment. The participants’ morphological characteristics showed no difference between the two groups (Table 1). DR athletes had training experience of 17.7 ± 3.50 years, with 9.6 ±2.36 years of practice in competition and a weekly activity of 32.88 ± 3.72 hours. Participant’s exclusion criteria included a documented balance disorder, a medical condition that might affect postural control, or a neurological/musculoskeletal impairment in the past 2 years.

**Table 1:** Mean characteristics of the DR and NA groups (standard deviation in parentheses) and statistics (t-test).

	Groups		Statistics		
	DR	NA	t-value	Levene F(1.31)	Levene p
Participants (n)	10	12			
Age (years)	24 (2.2)	22.33 (2.61)	1.25	0.29	0.60
Body mass (Kg)	56.5 (3.1)	59.33 (4.55)	-1.30	1.33	0.26
Body height (cm)	165 (0.03)	167.8 (0.03)	-1.05	4.44	0.05
Body mass index (kg.m^2^)	20.78 (2.03)	21.06 (1.53)	-0.38	0.67	0.42

All participants provided written informed consent and the study was approved by the ethical committee of the Science Faculty, Université Paris-Sud.

### Postural tests

Participants stood barefoot on a force platform (Medicapteurs, Fusyo model, “40Hz/16b”) with heel distant from 2 cm, with an external open angle of 30°, their hands hanging loosely by their sides and legs straight using the Standards of the Association Française de Posturologie.

**Fig 1:**
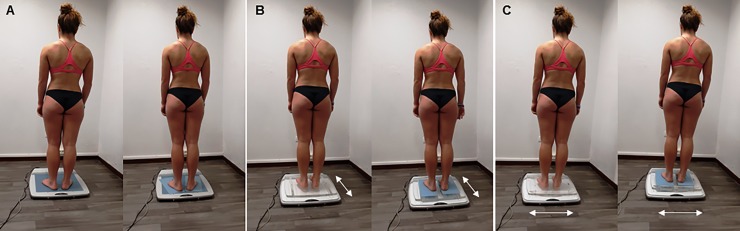
Experimental conditions and set-up: STA balance wF and noF (A); AP balance wF and noF (B); ML balance wF and noF (C). EO and EC conditions were recorded for each balance condition. The individual in this manuscript has given written informed consent to publish this figure.

Three balance conditions were investigated while participants were standing: (1) a static balance condition on a rigid floor (STA), (2) an unstable posture on a seesaw device generating instability in the anteroposterior direction (AP dynamic balance), and (3) an unstable posture produced by the seesaw device in the mediolateral direction (ML dynamic balance) (Fig 1). The seesaw device was 55 cm long and 6 cm tall (Bessou Dynamical Plate, Medicapteurs, France) [24]. Posture conditions were analyzed with eyes open (EO) and eyes closed (EC), and with a foam (wF) on the force platform (height: 0.2 cm, hardness: 8 SH, density: 220 kg.m^-3^) or not (noF). Each trial lasted 31.6s [25]. The order of the presentation of each trial was randomized. Each trial was conducted only once to avoid learning.

The force platform allowed measuring the displacement of the center of foot pressure (COP). Signals from the force platform were sampled at 40 Hz and filtered with a second-order Butterworth filter (8 Hz low-pass cut-off frequency).

### Data analysis

Four stabilometric parameters were used to describe the postural behavior of the participants:

-the COP surface (in mm^2^) which corresponds to the area of a 95% confidence ellipse and constitutes a measure of the CoP spatial variability;
- the mean COP velocity (in mm.s^-1^) which represents the sum of the cumulated COP displacement divided by the total time and constitutes a good index of the amount of activity required to maintain stability [26];
- the VFY parameter which has been obtained by dividing the rectified standard deviation velocity by the mean position on the AP axis. This parameter would help monitoring short length-high velocity compensating movements used to maintain the upright position [27–29];
- the Romberg Quotient (RQ) which corresponds to the relation between the COP surface parameters in EO and EC conditions on hard and foam ground.

RQ=(COPSurfaceEC/COPSurfaceEO)×100 (1)

### Statistical analysis

The statistical significance threshold was fixed at 0.05 (Statistica, StatSoft, USA). A Shapiro test and a Levenne test were performed on data to verify the normality of the data and the homogeneity of the variances, respectively. Then, a repeated measures analysis of variance (ANOVA) with 3 factors was carried out: 2 Groups (DR vs NA) x 2 Vision conditions (EO vs EC) x 2 Foam conditions (wF vs noF). The three balance conditions (STA, AP, ML) were independently analyzed. Newman-Keuls post-hoc was used to test differences among means. As the RQ included EC and EO visual conditions, postural quotients were specifically tested with a t-test, for each foam condition.

## Results

We conducted separate 2 Groups (DR vs NA) x 2 Vision conditions (EO vs EC) x 2 Foam conditions (wF vs noF) ANOVAs with repeated measures on the two last factors (Table 2). In order to address our main hypotheses with conciseness, we described the results of these different ANOVAs together for each main effect and each interaction in the next paragraphs.

**Table 2:** Level of Group × Foam × Vision ANOVAs main and interaction effects conducted on COP surface, COP velocity, VFY for the three balances (STA, AP, ML). Significant differences are indicated in bold (p<0.05).

	Group		Foam		Vision		Foam × Group		Vision × Group		Foam × Vision		Foam × Vision × Group	
	F	p	F	p	F	p	F	p	F	p	F	p	F	p
**STA balance**														
COP Surface	0.342	0.565	1.556	0.227	3.488	0.077	2.648	0.119	0.044	0.836	1.206	0.285	0.049	0.827
COP Velocity	0.275	0.606	0.807	0.380	**39.641**	**0.000**	3.070	0.095	2.079	0.165	0.018	0.895	0.857	0.366
VFY	3.321	0.083	1.627	0.217	1.263	0.274	0.005	0.944	0.867	0.363	0.469	0.501	0.004	0.953
**AP balance**														
COP Surface	0.346	0.563	0.030	0.864	**14.498**	**0.001**	0.100	0.755	2.244	0.150	0.104	0.750	0.269	0.610
COP Velocity	0.017	0.898	**12.673**	**0.002**	**83.579**	**0.000**	1.212	0.284	3.104	0.093	0.002	0.963	0.365	0.553
VFY	1.134	0.300	**5.568**	**0.029**	**30.171**	**0.000**	**6.822**	**0.017**	0.507	0.485	0.097	0.759	2.042	0.168
**ML balance**														
COP Surface	0.074	0.788	1.285	0.270	**94.107**	**0.000**	1.468	0.240	0.352	0.559	1.155	0.295	0.903	0.353
COP Velocity	0.167	0.687	**15.265**	**0.001**	**166.520**	**0.000**	0.684	0.418	0.827	0.374	0.634	0.435	0.439	0.515
VFY	**5.679**	**0.027**	**5.855**	**0.025**	**35.945**	**0.000**	1.837	0.190	0.077	0.784	1.874	0.186	**6.115**	**0.023**

### Influence of group

**Fig 2:**
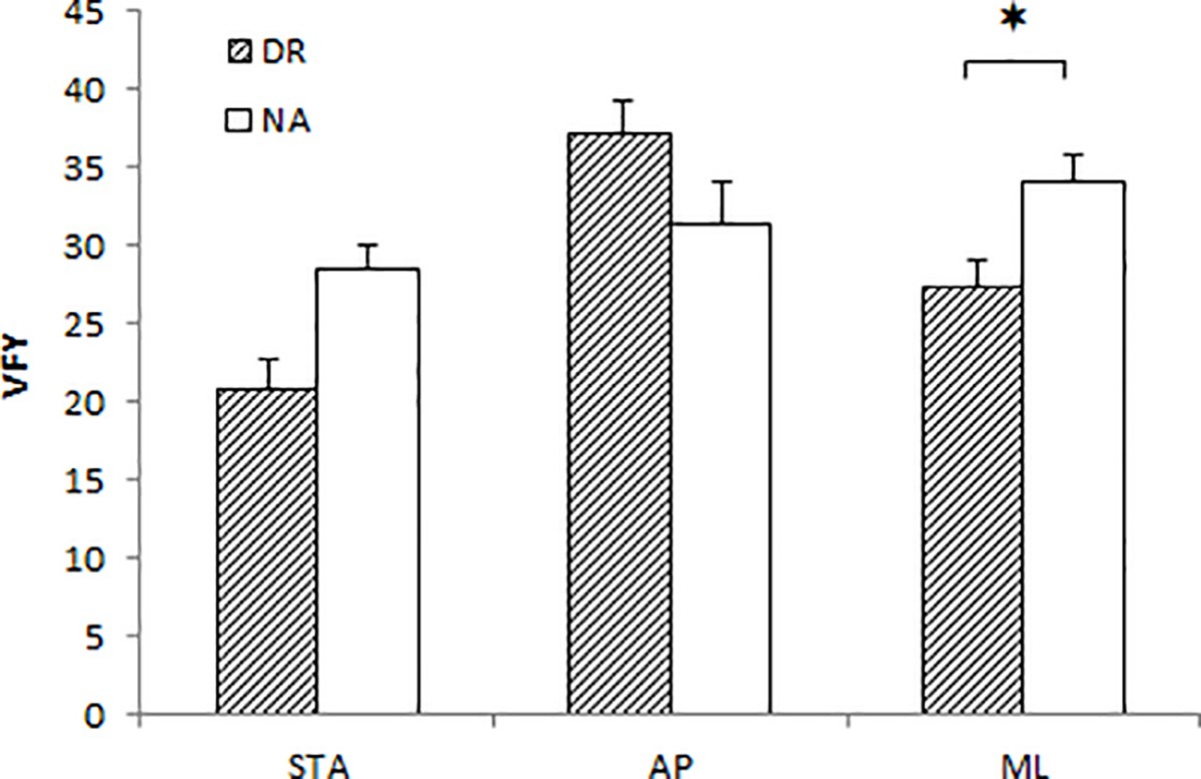
VFY for DR and NA groups across the three balances. Data are presented as mean and standard error. (*p<0.05).

In the STA balance and the AP dynamic balance condition, there was no significant effect of group on the COP surface, COP velocity, and VFY (see Table 2). Conversely, in the ML posture, VFY has been found to be significantly lower for DR than for NA (P<0.05) (Fig 2).

### Influence of vision conditions

In the STA balance, a significant main effect of the vision condition was found for the COP velocity only (see Table 2). Post-hoc test showed that COP velocity was significantly higher during EC condition (13.46 ± 0.66 mm/s) than during EO condition (9.81 ± 0.39 mm/s).

In the AP posture, COP surface, COP Velocity and VFY have been found to be significantly different through EO and EC conditions. Post-hoc tests revealed that: COP surface was significantly lower in EO condition (410.53 ± 34.37 mm) than in EC condition (1261.65 ± 181.66 mm), COP velocity was significantly lower in EO condition (20.26 ± 0.82 mm/s) than in EC condition (42.57 ± 2.27 mm/s), and VFY was significantly lower in EO condition (25.28 ± 1.89 mm/s) than in EC condition (42.75 ± 3.31 mm/s).

For the ML posture, a significant main effect of vision on all parameters was found. Parameters obtained during the EO condition were significantly lower (COP Surface: 402.38 ± 25.18 mm; COP Velocity: 21.22 ± 0.74 mm/s; VFY: 25.32 ± 1.59) than during the EC condition (COP Surface: 1390.85 ± 91.17 mm; COP Velocity: 42.85 ± 1.52 mm/s; VFY: 36.77 ± 1.65).

### Influence of foam condition

The STA balance revealed no significant effect of the presence of foam on COP surface, COP velocity, and VFY (see Table 2).

In the AP posture, COP velocity and VFY were significantly different between foam conditions. More precisely, the presence of foam on the force platform significantly decreased COP velocity (wF: 29.01 ± 2.63 mm/s; noF: 33.81 ± 2.10 mm/s) and VFY (wF: 31.17 ± 2.95; noF: 36.85 ± 3). Conversely, the main effect of foam was not significant for COP surface.

Same observation can be made for the ML posture, with COP velocity and VFY being significantly lower with foam (COP velocity: 29.01 ± 2.63 mm/s; VFY: 31.17 ± 2.95) than without (COP velocity: 33.81 ± 2.10 mm/s; VFY: 36.85 ± 3) and this main effect of foam did not reach significance for COP surface.

**Fig 3:**
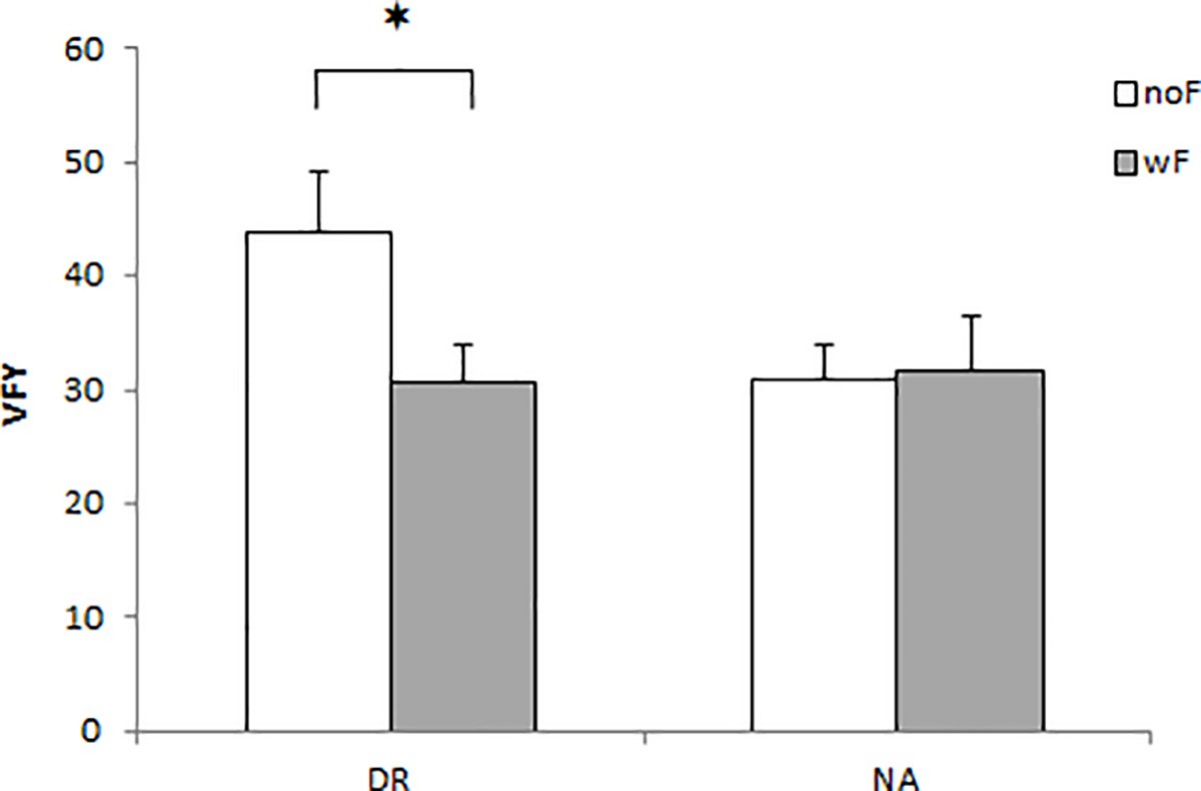
VFY in the AP dynamic balance with (wF) and without foam (noF) for DR and NA groups. Data are presented as mean and standard error (*p<0.05).

A significant interaction effect Foam × Group was observed on VFY in the AP posture (F_(1, 20)_ = 6.822, p<0.05). More precisely, post-hoc tests showed that the presence of foam in the DR group led to a significant lower VFY than without foam (p<0.05). No significant differences were found on NA group between the two foam conditions (Fig 3).

**Fig 4:**
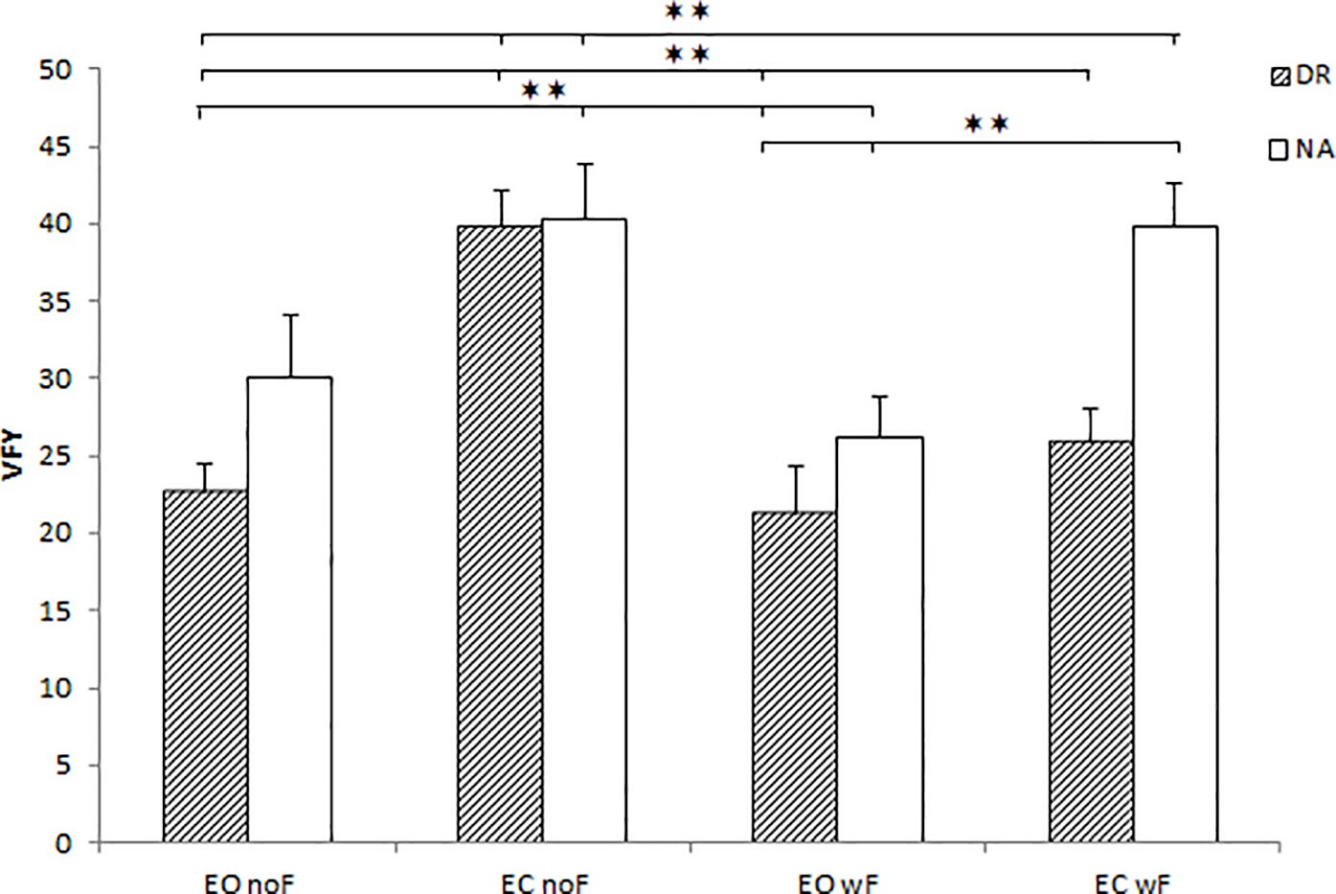
VFY in the ML dynamic balance (interaction effect) during EO and EC conditions (with and without foam) for DR and NA groups. Data are presented as mean and standard error (*p<0.05).

Finally, the ANOVAs conducted on COP surface, COP velocity showed no interaction effect on each balance condition (STA, AP, ML). However, the analysis conducted on VFY indicated that the Group × Foam × Vision interaction was significant (see Table 2) on ML dynamic balance. We found that in Eye Open no Foam condition was significantly less variable than in Eye Closed no Foam condition for DR (p<0.001), and in the same condition for NA (p<0.01). For NA, in Eye Open no Foam condition we did not found significant differences with other conditions (NS). Moreover, Eye Closed no Foam condition revealed a significant difference with Eye Open with Foam (p<0.001) and Eye Closed with Foam (p<0.01) for DR (Fig 4).

### Vision dependence

**Fig 5:**
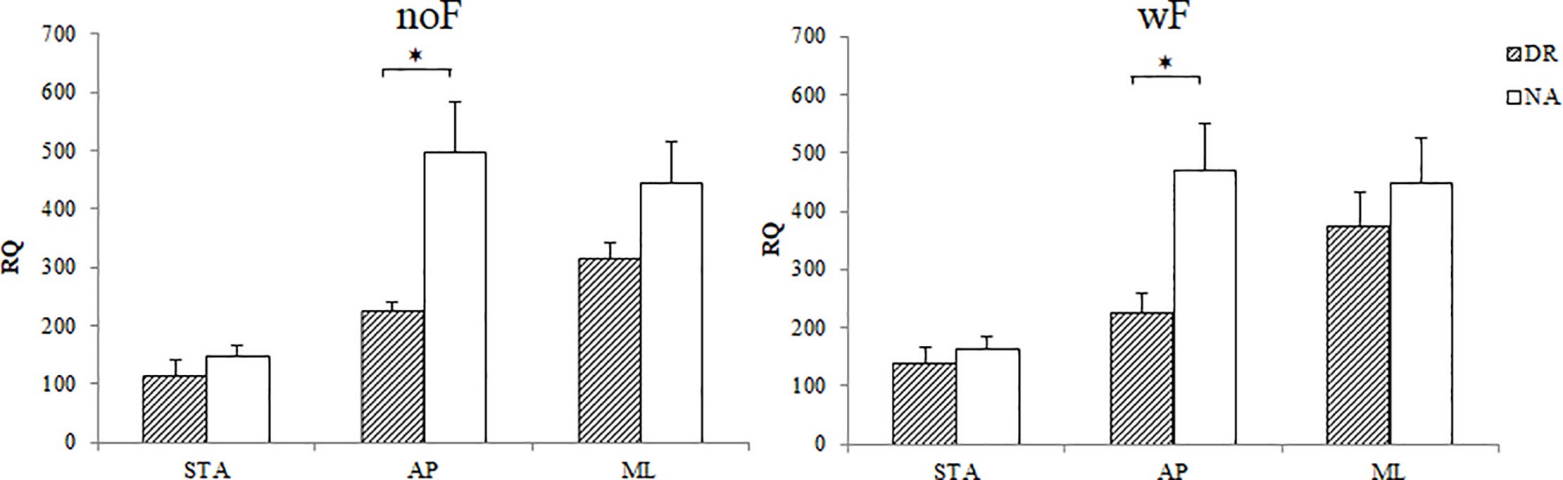
Romberg Quotient (RQ) for DR and NA groups for each of the three balances (STA, AP, ML) on COP surface (with and without foam). Data are presented as mean and standard error (*p<0.05).

The t-test analysis revealed that RQ was not significant in STA and ML postures between DR and NA groups (Table 2). However, in the AP posture, results showed significant differences between groups, RQ being significantly lower for DR group compared to NA group (Fig 5).

## Discussion

Horseback riding can be considered as a sport activity with particular postural constraints. The objective of this study was to analyze postural control of expert horse riders vs. non-athletes, in different visual and somesthesic conditions during static and dynamic standing balances. To achieve this goal, twenty-two young healthy adults, divided into two groups (DR and NA), were asked to stand upright during two visual conditions (EO and EC) and two somesthesic conditions (wF and noF). Centre of pressure (COP) displacements were recorded using a force plateform. Main results showed significant effect of groups on VFY during an unstable ML balance. This conventional parameter has been used by clinical posturographic practitioners to evaluate the importance of muscle contractions in relation to bipedal postural control, as it is known to capture the phenomenon of stiffness in the inverted human pendulum model [28,30]. Indeed, in an elderly population, an increase of the VFY parameter indicated a progressive reduction in the tension of the tissues of the posterior chambers of the legs [27].

Regarding the COP surface and COP velocity, our results showed that DR and NA exhibited similar values in STA, AM and ML balances. STA balance is a simple postural task which does not permit to discriminate the athlete postural ability [8,31,32]. However, in the ML dynamic balance, VFY was significantly lower for DR as compared to NA group. In other words, this postural parameter would appear as much more discriminating than traditional parameters (COP surface, COP velocity). This original outcome suggests that DR group had a better upright postural control than the NA group during ML dynamic balance. As in kayaker athletes, the dressage riders are part of the “upper-body sport” in “sitting posture” which differs biomechanically from other athletes studied in other postural stability investigations [5]. In horseback riding, there are two athletes, one human and one horse. The expert rider follows the motion of the horse’s body in order to optimize his interactions with the horse at different gaits. This synchronization with the horse implies having the ability to adapt balance and orientation to coordinated rider’s pelvis, trunk, head and limbs [13,16,33]. This sport-specific ability suggests that the rider develops specific muscles, such as the rectus abdominis and the erector spinae to stabilize the trunk, the adductor muscles to maintain the knee and the pelvis stability [34,35]. It may be proposed that riders would develop a greater ability to monitor short length-high velocity, thus compensating movements used to maintain the upright position.

Indeed, it can be suggested that the repeated movements of the pelvis and stresses on the spine during horseback riding practice would make horse riders more efficient when represented into an inverted pendulum model relative to the support of the saddle. The saddle would then represent the main surface of support as well as stirrups. Action and reaction mechanisms of the center of mass in relation to the COP would feed into the idea of anticipatory mechanisms experienced by horseback professional riders. These mechanisms have been reported while analyzing kinematical phases of riders on an equestrian simulator [13], or in an ecological environment with the horse [16]. Thus, a perspective of the current work would be to measure same postural parameters while sitting on a saddle, i.e. a straddle posture, in order to analyze the influence of posture specificity. Again, this ecological posture might be obtained using an equine simulator or directly on a horse. An intermediary protocol would be to assess postural stability in a standardized environment while sitting, as in [36].

Postural tonus has been shown to be more developed on the axis of displacement related to the sport-specific environment [5,37]. Knowing that horseback riding practice has been defined as an "interactive" dynamics which solicits the muscles of the trunk in a sagittal and vertical axes [15,38], as much as other sports such as judo [39], or gymnastics [32,40], it can be suggested that the influence of horseback riding would be better expressed in ecological situations.

An interesting finding of this study was the contribution of visual information. In EC condition, the COP surface, the COP velocity and the VFY were higher than in eyes-open conditions for two unstable balances (AP and ML balances). For the static balance (STA), a significant difference has been found only on COP velocity. We hypothesized that dynamic balances (AP, ML) induced more visual flow than a static balance, which help discriminating sensory information. Indeed, previous studies on other sport activities revealed an increase of the COP displacement during EC condition [8,10]. This assumption is based on the traditional approach which states that postural control aims to immobilize the center of mass despite movement and external perturbations [41–44]. However, based on Gibson’s work [45], an ecological approach of postural control with both theoretical and empirical supports also exists. This approach states that there is no relative weighting of sensory information rather all senses provide information that increases specificity in postural control [46,47]. Thus, as weighting of sensory inputs were not directly measured in the current study, it can be noted that our results do not exclude any theory of sensory perception.

According to the traditional approach, horseback riders would show less visual dependency than non-athletes during an AP dynamic balance on hard and soft ground. This adaptation would result from their equestrian practice. The sitting practice of professional riders might therefore lead to a specific reweighting when analyzing the bipedal standing posture. To better investigate the influence of practice on postural control, a follow-on study will be conducted with non-athletes, expert horse riders and experts from another sport practice.

Another interesting finding concerned the influence of foam support. Traditionally, posturographic studies investigated visual conditions (EO and EC), but less frequently with foam under the feet. However foam could have a key role during static balance to compensate the destabilization created by an EC condition on postural parameters. Indeed, into a healthy population, some participants appeared more sensitive to somesthesic information [48,49]. This can be explained by the fact that plantar elements of the foot are first points of contact between body and the external environment while standing, thus providing detailed spatial and temporal information about contact pressures under the foot and shear forces resulting from body movement [50–52]. Since cutaneous feedback from the plantar surface may be influenced by the interaction of the foot with the ground, it has been found that changing the characteristics of the supporting surface in a repeated manner modified the control of bipedal posture [53][54].

Again, it is interesting to note that the foam condition especially in eye closed condition was not different than eye open condition and was different than the eye closed in no foam surface only on the dressage rider. This original result of the interaction suggested that the dressage rider used somesthesic plantar information as their “eye”. Previous studies reported that expert in sport could shift the sensorimotor dominance from vision to proprioception for postural maintenance [8,9,55]. In the absence of visual information and when the dressage rider was in ML dynamic balance, the foam increased their balance and cancels the effect of eyes closed. As has already been proved by previous studies for similar activities practiced on unstable support (surfers [10]; kayakers [5]), it may be suggested that sport practitioner would show a lower dependence on vision for postural control. In fact in horseback riding, various contacts (with saddle, rein, stirrup, for example) and pressures (between the rider pelvis and the horse saddle, essentially), are produced during the horse/rider interaction in horseback riding. They provide rich and patterned somesthetic information (proprioceptive and tactile) that are of first importance for the rider to regulate and coordinate his/her movements with those of the horse. Dressage rider group was professional and they rode horses every day (35 hours by week). Therefore, these information (proprioceptive and tactile) help the rider to anticipate the horse movement as in our dynamic equilibrium test which was probably the closest condition to practice.

A limitation of this study may come from the fact that only women participants have been examined. This selection has been done to prevent a potential bias related to the influence of gender on postural parameters, although there is no real consensus about gender effects on postural stability in the literature. One of the first studies about this topic revealed no difference between six postural control measures between men and women [56]. Steindl and colleagues [57] investigated the development of sensory organization according to each sensory component (proprioceptive, visual, and vestibular) in relation to age and gender. They detected no gender difference in the adult group, as well as other studies from the literature [58,59]. However, Ericksen and Gribble [60] assessed dynamical postural control in men and women through the posteromedial reaching distance. They demonstrated that women presented significantly less dynamical postural control than men. In perspective, a follow-on study will compare these two groups of participants to male non-athletes and male dressage riders to investigate the influence of gender.

## Conclusion

Very little research has been devoted to the use of sensory information in horse riding and, none has been specifically devoted to the contribution of sensory information to upright postural stability. The aim of this study was to assess postural control differences between a group of horseback riding women (DR) and a group of non-athlete women (NA). First, compared to non-athletes, horseback riders exhibited greater VFY stability during a ML dynamic balance. Secondly, with foam on the ground during an AP dynamic balance, horseback riders revealed better stability than non-athletes. Thirdly, horseback riders showed less visual dependency than non-athletes during an AP dynamic balance. Thus, COP surface and COP velocity was not easy to discriminated the dressage rider to the non-athlete upright posture ability. The use of the VFY allowed us to show differences between groups.

## Supporting information

S1 File Dependent variables recorded through the force platform: Surface area covered by the CoP (mm^2^), Mean velocity of CoP (mm/s), VFY, Romberg Quotient of Surface area in different balance conditions: stable standing posture, unstable standing postures (AP seesaw and ML seesaw); and in different sensory conditions: Vision, No Vision, No Foam, Foam. (XLSX)
